# mRNA- and Adenovirus-Based Vaccines against SARS-CoV-2 in HIV-Positive People

**DOI:** 10.3390/v14040748

**Published:** 2022-04-01

**Authors:** Anna Rosa Garbuglia, Claudia Minosse, Paola Del Porto

**Affiliations:** 1 Laboratory of Virology, “Lazzaro Spallanzani” National Institute for Infectious Diseases, IRCCS, 00149 Rome, Italy; claudia.minosse@inmi.it; 2 Department of Biology and Biotechnology ‘C. Darwin’, Sapienza University, 00100 Rome, Italy; paola.delporto@uniroma1.it

**Keywords:** SARS-CoV-2, mRNA vaccine, adenoviral vaccine, HIV infection, immune response

## Abstract

About two years have passed since the identification of SARS-CoV-2 in China. The rapid spread of this virus all over the world and its high transmissibility and pathogenicity in humans have resulted in a global pandemic. The negative impact of COVID-19 on health, society and the economy at the global level has pushed researchers and pharmaceutical companies to develop effective vaccines to fight SARS-CoV-2. Thanks to this collaborative effort, the first COVID-19 vaccine was developed in less than a year. Since then, several COVID-19 vaccines have been validated for use by the World Health Organization. Among these, mRNA- (BNT162b2 and mRNA1273) and adenovirus-based (ChAdOx1) vaccines were developed through the use of novel technologies. While all three of these vaccines have shown effectiveness against the COVID-19 disease and their immunogenicity was characterized in clinical trials in the general population, data on their efficacy and immunogenicity in people living with HIV (PLWH) are limited. In this review, we provide a description of the characteristics of mRNA- and adenovirus-based vaccines and of the immune response elicited in the general population by vaccination. Then we describe the use of these vaccines and their efficacy and immunogenicity in people living with HIV and we conclude with a discussion regarding some open questions concerning the use of mRNA- and adenovirus-based COVID-19 vaccines in PLWH.

## 1. Introduction

The SARS-CoV-2 virus appeared in Wuhan (China) in December 2019 and it quickly spread to all the continents. The WHO estimates that by January 2022, approximately 380,000,000 people had contracted the SARS-CoV-2 infection, and about 5,700,000 people had died from this virus [1]. The rapid spread of the virus and its high transmissibility and high mortality rates, which have mostly been observed in fragile people such as the elderly, cardiopathic patients and the immunosuppressed, have highlighted the need to develop vaccines very quickly. Several approaches have been used, including developing vaccines based on recombinant proteins and on the live and attenuated viruses that were already available in China. However, vaccines based on either mRNA technology or on adenoviral vectors have, without doubt, been the most widely used. This is due to the speed with which they can be developed and the quantity of doses that can be obtained in a short time. However, people have been wary of both mRNA and adenoviral vector vaccines, which were the first to be used on a large scale, in fragile populations and without adequate comprehensive information on potential long-term adverse health effects. This has created hesitancy in accepting these vaccines. In this review, after a description of the characteristics of the adenoviral vector and mRNA vaccines, we describe their use in HIV subjects (PLWH), their efficacy and immunogenicity and the questions that have yet to be clarified.

## 2. Adenovirus Vaccines

The adenovirus (AdV) belongs to the family of Adenoviridae, genus Mastadenovirus. To date, 103 human adenoviruses (HAdVs) have been identified [2]. They are grouped in seven species (AdV A to AdV G) based on phylogenetic analysis, oncogenicity and genomic organization [3,4]. AdV is a non-enveloped virus with an icosahedral capsid and a linear double-stranded DNA genome, the size of which ranges from 26 to 45 kilobases (Kb) [5]. It encodes ~40 proteins, which are classified as “early” and “late” proteins. The early protein genes E1A, E1B, E2 and E4 are expressed before DNA replication. The late genes (L1–L5) encode for the penton base, hexon and fiber that constitute the capsid structure. Moreover, late genes encode “core” proteins (i.e., protein VII, protease). The life cycle of the virus, which includes DNA replication, gene expression and virion formation, occurs in the nucleus. Therefore, the risk of genome integration exists, but the vector predominantly remains episomal [6]. Even though the human adenovirus (HAdV) receptor varies among the species, the coxsackievirus and adenovirus receptor (CAR) represents the main route for viral entry into the host cell [7,8]. CAR expression in many cellular types confers a wide tropism to AdVs. Indeed, hepatocytes, myoblasts and epithelial and endothelial cells, which all have CAR receptors, are permissive to AdV infection. AdV species B, C and E are the most frequently associated with upper respiratory tract infections. Species C (types 1, 2 and 5) is responsible for mild infections in both children and adults. It has been estimated that ~5% of respiratory infections in children are caused by HAdVs, and they often lead to bronchitis or pneumonia, which require hospitalization [9,10]. Different species can be associated with different pathological manifestations. Species F and G are linked to infections of the gastrointestinal tract, while species B, D and E can also infect the conjunctiva. In particular, the HAdV 8, 19, 37, 53, 54 and 56 types, belonging to the D species, are the main agents of epidemic keratoconjunctivitis (EKC), which is endemic in Japan [11]. The frequent occurrences of outbreaks of infections caused by different HAdVs have favored viral mutations and recombination events [12]. One of the emergent strains is Ad14p1, a member of the B species which is still circulating and continues to cause outbreaks of influenza-like illness (ILI) [11,13]. It has been observed that 17% of patients infected with this serotype require intensive care support and mortality reaches 5% [14,15]. In general, adenovirus infection is asymptomatic. Symptomatic forms can involve a specific organ (the ophthalmic apparatus, respiratory tract, or gastrointestinal tract) or they can provoke disseminated disease, affecting lung, hepatobiliary systems, the genitourinary tract and gut [16]. Viral persistence can be associated with the presence of adenovirus in tonsillar or intestinal lymphocytes [17,18,19]. AdV infection is prevalently observed in the first years of life, and in some cases, AdV infection of the respiratory tract results in hospitalization [9,10]. However, fatal outcomes in immunocompetent individuals are rare and have been primarily reported in cases of pneumonitis [20]. The severe infections occur in immunocompromised patients, including HIV-positive patients [21,22]. T-cell depletion constitutes a key risk factor for AdV infection and the capacity to generate adenovirus-specific CD4+ T cells plays a crucial role in the evolution of the infection [23]. In cardiac transplant patients, AdV infection has been reported to be one of the main causes of late or chronic rejection [24]. In a cohort of transplanted adults, 27% of subjects who contracted AdV infection after transplantation died of AdV-related diseases, especially hepatitis and pneumonitis [25]. The cause of the severity of AdV infection in these patients remains unclear. It has been speculated that the depletion of T and NK cells, combined with the decrease in interferon-γ production subsequent to corticosteroid and chemotherapy treatment, might be relevant in this phenomenon [26,27].

### Adenovirus Vectors

Adenoviruses have been chosen as viral vectors due to their ability to stably express inserts of up to 8 kb to generate high numbers of progeny virions in in vitro systems and for their ability to evoke a robust adaptive immune response without adjuvant compounds, which therefore simplifies vaccine composition [28]. The adenoviruses used as viral vectors in human vaccines are unable to replicate (replication deficient, RD), thus they do not produce infectious viruses. In these viral vectors, several viral genes are deleted. Generally, the E1A and E1B (early transcript 1A and 1B) genomic regions are replaced by an antigen expression cassette, thereby abolishing the viral ability to replicate [29]. The E3 gene is also frequently deleted to prevent the elimination of AdV by the immune system, while E4 is removed to facilitate the expression of the inserted antigen [29]. Adenoviral vectors maintain only the left and the right inverted terminal repeats (ITR) and packaging signal of the AdV genome [3]. Adenoviral vectors are able to induce potent B- and T-cell responses, even though their magnitude depends on the viral genotype employed [30]. For example, AdV type 5 is the first and most used adenoviral vector because it is able to induce exceptionally high numbers of CD8+ T cells as well as strong antibody responses [31]. Nevertheless, human adenoviral (HAdV) vectors have limited use in human vaccinology because of the pre-existing immunity in the general population. Thus, AdVs are ubiquitous and infect the majority of the population. For example, the seroprevalence of AdV5 and AdV55 was 60% and 7%, respectively, in Dutch people at risk of AIDS, while the seroprevalence of the same adenoviruses reached 90% and 20%, respectively, among people living in Sub-Saharan Africa [32,33]. For this reason, nonhuman adenoviral vectors are often used for vaccine development and include those that infect the Rhesus macaque (RdAd63), chimpanzee (ChAd), gorilla (GAd) or rarely humans, such as genotypes AdV26 or AdV35. Immunization with AdV26 and AdV35 preferentially elicits more long-lived central memory CD8+ T-cell responses than AdV5 [30]. Nonhuman genotypes are genetically and structurally similar to human AdVs, making them easily adaptable as vaccine vectors. Replication-deficient, third-generation vectors with the deletion of the E1 and E3 genes have been engineered for the expression of SARS-CoV-2 spike protein, which contains the major epitopes recognized by neutralizing antibodies [34]. The SARS- CoV-2 vaccines are based on different AdV types containing the spike (S) protein, which is considered the major antigen target for human anti-coronavirus vaccines [35]. Several AdV vectors have been used in SARS-CoV-2 vaccines. For example, the CanSinoBiologica vaccine contains an AdV5 viral vector, the Johnson and Johnson vaccine is based on HAdV26 viral vector and administered in a single dose, the Russian SPUTNIK vaccine is based on prime-vaccination with AdV26 viral vector-SARS-CoV-2, followed by a booster vaccination with AdV5-SARS-CoV-2, and AstraZeneca used a chimpanzee vector, ChAdOx1nCoV-19 [36,37,38,39,40]. Before administration to the general population, all clinical trials were carried out on HIV-negative subjects aged <60 years.

## 3. mRNA Vaccines

mRNA vaccines play an important role in current vaccinology for several reasons:

1. The speed of their development. In fact, it usually takes >10 years to develop a conventional vaccine. Conversely, the SARS-CoV-2 vaccine Moderna mRNA-1273 was prepared in only 42 days following the online availability of the SARS-CoV-2 spike-protein coding sequence in GenBank, and after 10 months, the vaccine was authorized for emergency use by the FDA [41];
2. They are not infectious;
3. The mRNA is rapidly delivered into the host cell cytoplasm by lipid nanoparticles (LNP). Shortly after protein translation, it is degraded by cellular enzymes;
4. mRNA vaccines are able to induce both humoral and cell-mediated immunity, stimulating potent MHC-class-I- and MHC-class-II-restricted T-cell responses;
5. The mRNA vaccine does not stimulate adaptive immune responses, thus no pre-existing immunity can interfere with the efficacy of the vaccine after the booster doses;
6. Both mRNA and LNP have adjuvant properties.

The synthesis of mRNA comprises several steps:

1. Generally, a linear plasmid or amplicon is used as a template for RNA synthesis. The most commonly used RNA-polymerases are T3, T7 or SP6. The translational efficiency is improved by codon optimization and nucleoside modification (generally pseudouridine replaces the uridine). The activation of TLR-3, -7 and -8 is abrogated by introducing pseudouridine in the mRNA or m5C, m6-A, m5U or s2U [42,43]. Furthermore, the presence of pseudouridine m6A and s2U in the mRNA vaccine molecules hampers the degradation of RNA by cellular RNAse [44]. In all COVID-19 mRNA vaccines, uridine has been replaced by pseudouridine (m6A and s2U);
2. A 7-methylguanosine (m7G)5′ trisphosphate cap is added to the 5′ end to allow the recognition of the mRNA vaccine by cytoplasmic factors involved in the translation process [45]. This cap is able to eliminate free phosphate groups in the mRNA sequence, thus enhancing mRNA stability [45]. The 5′ cap represents a determining factor through which the host can discriminate between self- vs. non-self-mRNA. Moreover, anti-reverse cap analogs (ARCA) have been introduced to prevent the reverse incorporation of the 5′ cap [46,47]. ARCA is modified at the C2 or C3 positions to ensure that the methyl groups react with hydroxyl groups at the correct site during transcription, enhancing the translational efficiency [46,48]. Innovative protocols set up the addition of the 5′cap to a given start sequence during in vitro transcription [49,50]. Gene expression is enhanced by the untranslated (UTR) sequence addition to mRNA [51,52]. The 5′UTR shows a direct influence on translation of the downstream (sequence) open reading frame (ORF). Furthermore, some specific sequences can be added to the 5′UTR to strengthen the accuracy of translation and the stability of the mRNA [53,54]. The stability and the extension of the mRNA half-life are markedly increased by the 3′UTR sequence in the mRNA vaccine [55,56];
3. Polyadenylation tail poly (a) reduces the degradation of mRNA mediated by the RNA exonuclease, guaranteeing a great efficiency in translation [57]. The length of poly (a) is not absolute, and it depends on the cellular milieu where the mRNA is translated. A poly (a) sequence of over 300 nucleotides in length may be more effective in ensuring mRNA expression in primary T cells, while small poly (a) (120–150 nt in length) sequences were found to be optimal for mRNA expression in dendritic cells [58]. A poly (a) sequence which is shorter than 20 nt reduces mRNA translational efficiency in all human cell types [59]. However, the information on the 5′ and 3′UTR and poly (a) composition of the SARS-CoV-2 mRNA vaccines remains undisclosed and the intellectual property of pharmaceutical companies;
4. mRNA purification: the mRNA produced by in vitro transcription (IVT) should be purified before being incorporated into the LNP. In fact, the double-stranded RNA–RNA and DNA–RNA hybrid molecules can stimulate the innate immune response, which can weaken the effectiveness of the vaccine since the innate immune activation could provoke mRNA degradation and reduce the production of the immunogenic protein [60,61]. The main method employed for mRNA purification is high performance liquid chromatography (HPLC) [50,57]. In an alternative and cheaper method, the RNA–RNA hybrid molecules are adsorbed onto cellulose polysaccharide [61]. Nevertheless, the mRNA of COVID-19 vaccines is purified by HPLC.

### Delivery System

The main system to deliver the mRNA vaccine into the cellular cytoplasm is through ionizable lipid-containing particles (LNPs) [62,63].

They are composed of: (a) a bilayer of ionizable cationic lipids that encapsulate the mRNA at low pH; (b) natural phospholipids that support the bilayer structure; (c) cholesterol that stabilizes the lipid bilayer and helps fusion with the cellular membrane; and (d) lipid-anchored polyethylene glycol (PEG), which reduces nonspecific protein adsorption and increases the half-life of LNPs [64,65]. Cationic lipids have been associated with the activation of several cellular pathways, such as proapoptotic and proinflammatory cascades [66].

## 4. Immunogenicity of mRNA and Adenoviral-Vectored Vaccines against SARS-CoV-2 in People without HIV/Healthy Individuals

Currently approved vaccines against SARS-CoV-2 target the trimeric spike glycoprotein, which plays a major role in initiating viral infection via the interaction of its receptor-binding domain (RDB) with angiotensin-converting enzyme 2 (ACE-2) [67]. Among these vaccines, five have been authorized by the FDA and EMA, and they include the two mRNA vaccines BNT162b2 (Pfizer/BioNtech, Pfizer, Inc.; Philadelphia, PA, USA) and mRNA-1273 (Moderna, ModernaTX, Inc.; Cambridge, MA, USA), the two adenoviral-vectored vaccines ChAdOx1 nCoV-19 (AZD1222, University of Oxford/AstraZeneca, UK) and Ad26.COV2.S (Janssen, Janssen Biotech, Inc., a Janssen Pharmaceutical company, Johnson & Johnson; Horsham, PA, USA) and the protein-based vaccine NVX-CoV2373 (Novavax, Inc.; Gaithersburg, MD, USA).

Despite the proven clinical efficacy of these vaccines, the understanding of how they induce immune responses related to protection is still limited. Numerous studies have evaluated the antigen-specific antibody and T-cell responses induced by the mRNA and adenoviral-vectored vaccines. Much of our knowledge of the immunogenicity of these vaccines derives from the analyses of phase I/II and phase II/III clinical trials.

### 4.1. Adenoviral-Vector-Based Vaccines

The immunogenicity of the ChAdOx1 nCoV-19 vaccine (AZD1222) was first evaluated in a phase I/II single-blind, randomized, controlled trial in healthy adults in the United Kingdom [68]. A total of 1077 participants, aged 18–55 years were randomly assigned to receive either the ChAdOx1 nCoV-19 at a dose of 5 × 1010 viral particles (*n* = 543) or the MenACWY (meningococcal ACWY) (*n* = 534) vaccine as a single, intramuscular injection. Ten participants received a second vaccine dose 28 days after the first one [68].

Evaluation of the humoral immune response demonstrated that participants who received a single dose of the ChAdOx1 nCov-19 vaccine showed an increase in antibodies against the SARS-CoV-2 spike protein, which peaked by day 28 and remained elevated until day 56. After a booster dose, the antibody response markedly increased. Similarly, the two-dose regimen elicited higher titers of neutralizing antibodies (Nab) against SARS-CoV-2 in 100% of participants, while neutralizing antibodies in the single-dose group were detected in between 67% and 100% of participants, depending on the assay adopted [68].

The ChAdOx1 vaccination elicited high numbers of spike-specific IFN-γ-secreting T cells, as demonstrated by an ex vivo ELISpot assay with peripheral blood mononuclear cells. Adenovirus-vectored-vaccine-induced virus-specific T-cell responses peaked 14 days after vaccination and persisted, although at lower levels, until day 56 after vaccination [68].

In-depth analyses of the immunogenicity of the ChAdOx1 nCoV-19 in individuals recruited for the phase I/II clinical trial who received one or two doses of the vaccine were performed in two following studies [69,70].

Characterization of the cellular immune response in adults aged 18–55 years who received a single dose of ChAdOx1 nCoV-19 showed that IFN-γ-secreting T cells mainly directed against the S1 domain were elicited 14 days after vaccination. Antigen-specific T cells included high frequencies of CD4+ T cells secreting predominantly Th1 cytokines and monofunctional, polyfunctional and cytotoxic CD8+ T cells.

The same vaccination induced spike-specific IgM, IgA and IgG. IgG responses were predominantly composed of IgG1 and IgG3 and showed a progressive increase in avidity until 56 days after vaccination. Both the cellular and the humoral immune responses were similar in males and females [69].

Immunogenicity data from individuals who received a vaccine booster dose at a 28- or 56-day intervals demonstrated an increase in the IgG antibodies to the SARS-CoV-2 spike and receptor-binding domain after the second vaccination [70]. At 14 days after the second dose, no difference in the anti-spike titers could be detected between individuals who received the booster at day 28 or day 56.

Additionally, NAb, measured by a microneutralization assay (MNA) or a pseudovirus neutralization assay, increased after the second vaccination regardless of the interval.

ChAdOx1 nCoV-19 administration induced anti-spike IgM, IgA and IgG. IgM response peaked 28 days after prime, while IgG1 and IgG3 increased following booster vaccination. Antibodies induced by the first vaccination were able to support antibody dependent neutrophil/monocyte phagocytosis (ADNP, ADMP), complement activation (ADCD) and natural killer cell activation (ADNKA) and these functions increased after the second vaccination.

The second vaccination did not affect the magnitude of the spike-specific T-cell response. Moreover, the frequencies of spike-specific IFN-γ-producing cells peaked 14 days after the first vaccination and did not increase after the booster dose [70].

A single-blind, multicentre, randomized, controlled, phase II/III trial assessed the safety and immunogenicity of the ChAdOx1 nCoV-19 vaccine at two different doses in groups of adults aged 18–55 years, 56–69 years and 70 years and older in a one-dose or two-dose regimen with a 28-day prime–boost interval [71].

The vaccine induced a specific antibody response to the SARS-CoV-2 spike glycoprotein and RBD at 28 days after a single dose across all age groups. A clear booster effect was observed in individuals who received a second dose of the vaccine independently of dose regimen or age group. Neutralizing antibody responses after the second dose were elicited in 99% of participants, and the antibody titers were similar among all age groups. SARS-CoV-2-specific T-cell responses measured with ELISpot peaked 14 days after the prime vaccination and did not increase significantly after the boost vaccination [71].

### 4.2. mRNA Vaccines

The immunogenicity of BNT162b2 was initially evaluated in a placebo-controlled, observer-blinded, dose-escalation phase I trial (NCT04368728) that compared the safety and immunogenicity of BNT162b1, which encodes a secreted trimerized SARS-CoV-2 receptor-binding domain, and BNT162b2 which encodes a prefusion stabilized, membrane-anchored SARS-CoV-2 full-length spike protein, in healthy adults aged 18–55 or 65–85 years [72]. Both groups received doses of 10 μg, 20 μg or 30 μg of BNT162b1, BNT162b2 or placebo on a two-dose schedule with a 21-day interval.

To assess immunogenicity, SARS-CoV-2 serum neutralization assay and receptor binding-domain (RBD)-binding or S1-binding IgG direct Luminex immunoassays were performed before the administration of vaccine and placebo, and at 7 days and 21 days after the first dose, and at 7 days and 14 days after the second dose. In both younger and older adults, the two vaccine-candidate groups elicited similar SARS-CoV-2-neutralizing geometric mean titers which were similar to or higher than the geometric mean titer of a panel of SARS-CoV-2 convalescent sera. The highest neutralization titers were measured in samples obtained 7 days or 14 days after the second dose and, in general, the antibody response elicited by the vaccines was lower in those over 65 years of age compared with younger participants.

Moreover, since the antibody response elicited by the two vaccine-candidate groups was similar, but BNT162b2 had a milder systemic reactogenicity, particularly in older individuals, BNT162b2 was chosen for phase II/III clinical studies.

The immune responses induced by BNT162b2 were more extensively evaluated in the phase I/II trial (NCT04380701) performed in Germany. In this study, healthy adults of 18–55 years of age received a priming dose of 1, 10, 20 or 30 μg on day 1 and a booster dose on day 22.

S1- and RBD-binding IgG concentrations and SARS-CoV-2 neutralizing titers were assessed on day 8 and 22 after the first dose and on day 29, 43, 50 and 85.

In all dose cohorts, the GMC (geometric mean concentration) of S1-binding IgG peaked on day 29 and it remained higher than that observed in convalescent sera at day 85 [73]. Similarly, SARS-CoV-2 GMTs increased substantially at day 29 after the booster dose and after a decrease, they remained stable from days 43 to 85. Sera from vaccinated individuals were able to neutralize pseudo-viruses carrying SARS-CoV-2 S variants with single or multiple aa substitutions, although with different efficiencies.

Analysis of the T-cell response in 37 individuals vaccinated with 1, 10, 20 or 30 μg of BNT162b2 by ELISpot assay using pools of overlapping peptides representing the S1 protein demonstrated that at dose levels of 10 μg or higher, the majority of individuals showed a robust expansion of poly-specific CD4+ and CD8+ T cells 1 week after boost (day 29). The majority of CD8+T cells and a considerable fraction of CD4+ T cells secreted IFN-γ. High percentages of vaccine-induced CD4+ T cells secreted IFN-γ, IlL-2 or both, while S1-specific CD8+ T cells secreted predominantly IFN-γ. Pre-existing CD4+ or CD8+ T-cell responses were detected in a minority of vaccinated participants [73].

Assessment of the humoral immune response elicited by two 30 μg doses of BNT162b2 vaccine in 12-to-15-year-old healthy individuals demonstrated the noninferiority of neutralizing titers in younger participants as compared with 16-to-25-year-old individuals 1 month after the second dose [74].

The immunogenicity of the second authorized mRNA vaccine (mRNA-1273) that encodes the S-2P antigen, consisting of the SARS-CoV-2 glycoprotein with a transmembrane anchor and an intact S1–S2 cleavage site, was initially addressed in a phase I study. The study was conducted with 45 healthy adults aged 18–35 years who received 2 injections of the vaccine at a dose of 25 μg, 100 μg or 250 μg 28 days apart [75]. All patients developed binding antibodies to both full-length S-2P and receptor-binding domain after the first vaccination in a time- and dose-dependent manner. Within variance, antibody neutralization responses were detected in all patients after the second immunization at day 43 and the values of neutralizing activity were similar to those of convalescent sera.

Vaccination with 25 μg and 100 μg doses elicited S-specific CD4 +T-cell responses strongly biased toward expression of Th1 cytokines (tumor necrosis factor α > interleukin 2 > interferon γ) while CD8+ T-cell responses to S-2P were detected at low levels only after the second vaccination in the 100 μg dose group [75].

The extension of the phase I study to include 40 additional participants, who were 56 years of age or older, demonstrated that neutralizing-antibody responses in older individuals appeared to be similar to those previously reported among vaccine recipients between the ages of 18 and 55. Similarly, the vaccine induced a strong Th1 T-cell response in participants receiving a 100 μg dose of vaccine [76].

The analysis of the durability of the humoral immune responses revealed that vaccination with 100 μg of mRNA-1273 produced high levels of binding and neutralizing antibodies that remained elevated 3 months after the booster dose in both younger and older adults [77].

### 4.3. Safety

The two mRNA vaccines, BNT162b1 and mRNA-273, as well as the adenoviral-vector-based ChAdOx1 nCoV-19 vaccine, showed good safety and immunogenicity profiles in the clinical trials. The main adverse effects reported were fatigue, chills, muscle pain and fever [40,78]. Younger individuals (<55 years old) generally presented a higher incidence and intensity of side effects compared with older individuals (>55 years old) for both types of vaccines [79]. However, recent reports demonstrated that the incidence of venous thrombosis, disseminated intravascular coagulation and thrombocytopenia was higher in subjects that had been vaccinated with ChAdOx19 nCoV19 in comparison with the general population [80,81,82]. Concerning mRNA vaccines, they showed more pronounced side effects after the second dose of immunization and the severe adverse events included facial paralysis (Bell’s palsy), paroxysmal ventricular arrhythmia and leg paresthesia [83].

## 5. HIV and SARS-CoV-2

Epidemiological studies reported that 1% of SARS-CoV-2-positive PLWH are hospitalized and the prevalence of the SARS-CoV-2 infection in PLWH is similar to that described in the general population, while the studies describing the severity of SARS-CoV-2 in PLWH do not report univocal data [83,84,85,86]. In a study of a wide cohort of HIV-positive and HIV-negative people, PLWH showed a risk of mortality that was 2.9 times (95% CI 1.96–4.30; *p* < 0.0001) greater than HIV-negative subjects. The mortality for COVID-19 seemed to be more strictly linked to Black ethnicity, where the risk of mortality was 4.3 times higher in comparison with HIV-negative people. However, this study did not take into account the impact of CD4+ T-cell count or the ART therapy in the prevention of mortality from COVID-19. In fact, demographic characteristics, lifestyle associated factors, including BMI and smoking, and relevant comorbidities were considered but not therapy regimen, CD4+ T-cell count and HIV RNA viral load [87], and these two factors are the most important determinants of severity and poor outcome of any co-morbidity in PLWH.

In a single center in Madrid (Spain), among 51 PLWH who were SARS-CoV-2-positive, 28 of 55 needed hospitalization, 13 of 25 developed a severe disease and the mortality was twice as high in comparison with people of similar age in the general population [88].

In another multicenter study carried out in the United States of America (USA), 286 PLWH and COVID-19-positive subjects were enrolled, and 94.3% of the patients were under HAART. Lower CD4+ T-cell counts (i.e., <200 cell/µL) represented the main factor linked to the higher percentage of intensive care unit admissions, mechanical ventilation or death [89]. In agreement, Nomah and colleagues showed worse COVID-19 outcomes in PLWH with viremia and CD4+ T-cell counts of less than 200 cells/µL [90]. Furthermore, in a cohort in New York, a higher rate of hospitalization and mortality was observed in PLWH with COVID-19 in comparison with HIV-negative people [91]. Conversely, Nagarakanti and colleagues observed no statistical difference in the disease outcomes between PLWH and HIV-negative people. Their study was carried out at the New Art Beth Israel Medical Center with 23 PLWH that had been admitted for COVID-19 disease. The median age of patients was 59 years and all of them were under ART therapy. Three patients died. CD4+ T-cell counts and viral load were available only for two of them, and they had undetectable viral loads and CD4+ T-cell count of >400 cell/µL. In this study, a low CD4 count did not necessarily imply a worse course of the disease. In fact, two-thirds of patients with a CD4+ T-cell count of <200/µL were discharged without any clinical complications (intensive care unit, ICU, ventilation) [92]. However, due to the low number of subjects analyzed in this study, no conclusions can be drawn regarding the relationship between lymphopenia and COVID-19 mortality. According to Wang et al., the milder course of the disease in HIV-positive people with low CD4+ T-cell counts could be explained by the impairment of the immune system as a consequence of HIV CD4+ T-cell depletion. In fact, these patients showed a delayed IgM antibody response and significantly longer disease course [93].

In a cohort from South Africa, among 22,308 SARS-CoV-2/HIV-positive patients, 625 (2.8%) died [94]. COVID-19 death was associated with a CD4+ T-cell count of <200 cells/µL at admission and with tuberculosis infection.

Furthermore, a Spanish case-control study demonstrated that PLWH had a higher mortality rate (9.8% vs 3.4%) compared with HIV-negative subjects. However, deceases were mostly linked to comorbidities in PLWH, and they were not related to HIV RNA suppression, CD4+ T-cell count or ART therapy administration [84].

Overall, among PLWH, a consistent number of deceased patients had other comorbidities, such as diabetes (50%) or hypertension (42%), similar to HIV-negative people. Overall, COVID-19 mortality was 2.4 times higher among PLWH than in HIV-negative people independently from their HIV RNA viral load value or immune suppression status. 

The discrepancies observed in different studies may be related to several factors, including:

1. The age of the patients. For example, in the United States, many HIV-positive patients are over 50 years of age with cardiovascular diseases;
2. Obesity. This factor is certainly not negligible and it can worsen the course of the COVID-19 disease;
3. Cardiovascular problems;
4. Diabetes [95,96,97,98,99,100,101,102,103].

In the New York cohort, patients had two or three comorbidities, and this may explain a more severe course of the disease [89].

In the South African cohort, which comprised subjects under ART, it should be established how and whether comorbidities such as diabetes might have favored the increase in the number of deaths among PLWH [94].

## 6. SARS-CoV-2 Vaccines in HIV-Positive People (PLWH)

The data on the SARS-CoV-2 vaccine efficacy in PLWH are still preliminary. Generally, PLWH who are under ART and with undetectable HIV RNA levels in serum respond well to both AdV- and mRNA-licensed vaccines. Useful information on vaccine use in PLWH can be found on the CDC website [104]. Here we report the main trials carried out among the PLWHs.

### 6.1. AdV-Vector-Based Vaccines

The immunogenicity of the CHAdOx1nCoV-19 in people with HIV was evaluated in a single-arm, open-label vaccination sub-study within a larger phase II/III trial. A total of 54 participants aged 18–55 years were enrolled and received 2 vaccine doses 4–6 weeks apart. All participants were on antiretroviral therapy with undetectable plasma HIV RNA and their CD4+ T-cell counts were of more than 350 cells/µL [105].

Antibodies against the SARS-CoV-2 spike protein peaked 14 days after the second vaccine dose and were sustained at day 56. These responses were significantly higher in PLWH compared with healthy individuals. Measurement of the neutralization activity of antibodies by a focus reduction neutralization test (FRNT) 28 days after the prime and the booster dose (day 56) in a randomly selected subset of 15 participants showed an increase in the percentages of participants with neutralizing antibodies, which corresponded to 13% and 87% at days 28 and 56, respectively.

IFN-γ T-cell responses against the SARS-CoV-2 spike, monitored 14, 28, 42 and 56 days after vaccination, peaked at day 14 and were significantly higher than baseline at all time points analyzed. No differences in the frequencies of virus-specific IFN-γ secreting cells were observed between HIV-positive individuals and adults without HIV. Evaluation of the proliferative capacity of CD4+ and CD8+ T cells against the SARS-CoV-2 spike demonstrated 2 peaks (at days 28 and 42) in the proliferative CD4+ T-cell responses depending on the spike region recognized by T cells, while CD8+ T-cell proliferative responses peaked on day 28. The similar magnitude and persistence of the SARS-CoV-2 spike-specific humoral or cellular immune responses induced by vaccines in individuals with or without HIV suggested that no dose adjustment was needed for people with HIV on ART with CD4+ T-cell counts of more than 350 cells per µL.

Moreover, since the people included in this trial had a sustained CD4+ T-cell count, no reliable data can provide indications about the protective action of the vaccine in people with low CD4+ T-cell counts or in those who are not under ART, who represent the largest percentage on the African continent, where more than 2 million PLWH are not under any therapy regimen [106].

A double-blind, placebo-controlled phase IB/IIA trial assessed the safety and the immunogenicity of the ChAdOx1 nCoV-19 vaccine in people with HIV in South Africa. Participants were adults aged 18–55 years and included 104 people with HIV and 56 HIV-negative individuals. PLWH were stable on ART for at least 3 months with a median CD4+ T-cell count of 695 cells/µL; 75% of participants had a viral load <50 copies/µL. Primary immunogenicity analyses in 44 HIV-negative individuals and 62 people with HIV who received two doses of ChAdOx1 nCoV-19 vaccine or a placebo 28 days apart demonstrated that immunized participants showed a strong serum IgG response against the full-length spike (FLS) and RBD proteins. In particular, the concentrations of either FLS IgGs or RBD-binding IgGs were similar in HIV-positive and HIV-negative individuals at day 28 after priming and they increased after the booster dose. Seropositivity for protein FLS and RDB were comparable in PLWH and HIV-negative people. In addition, people with HIV who were SARS-CoV-2 seropositive at baseline showed higher antibody responses after each vaccine dose compared with people with HIV who were seronegative at baseline [107].

### 6.2. mRNA Vaccines

The humoral immune response to mRNA vaccines was first assessed in a small study that included people with HIV (PWH) ≥18 years old in the US.

Total antibodies (IgM, IgG) to SARS-CoV-2 S-RDB were measured via the Roche Elecsys® anti-SARS-CoV-2 enzyme immune assay between days 17 and 27 after the first dose vaccination. The participants had a median age of 64 years, they had been under ART for at least 6 months and 92% had undetectable viral loads. They had CD4+ T-cell counts ranging from ≥500 µL to <200 cells/µL. Half of the participants received mRNA-1273 (Moderna) and the others received BNT162b2 (Pfizer). All participants developed anti-SARS-CoV-2 antibodies, although their levels were lower in the participants with CD4+ T-cell counts of <200 cells/µL [108].

The total antibodies (IgM, IgG) to SARS-CoV-2 S-receptor-binding domain (RDB) were also evaluated after the booster dose for the two mRNA vaccines in 12 PLWH.

Six participants received the Pfizer/BioNTech BNT162b2 vaccine and six received the Moderna mRNA1273 vaccine. All participants developed high titers of anti-RDB antibodies at a median time of 29 days after the booster dose. Plasma antibody titers of all participants were comparable to those seen in the immunocompetent HIV-uninfected population, apart from one individual with CD4+ T counts <200 cells/µL [109].

The humoral immune response to the Pfizer–Biontech BNT162b2 vaccine after the second dose in people living with HIV was evaluated in a study of 143 PLWH aged >18 years and 261 immunocompetent health-care workers (HCW) who received a booster dose at an interval of 21 days [110]. At the time of vaccination, the PLWH were on ART and 95% of them had an undetectable viral load, with baseline geometric mean CD4+ T-cell counts of 700 cells/µL. The average time of HIV diagnosis was 13 years and 26 had AIDS.

139 of 143 PLWH and 258 of 261 HCW developed RBD-IgG antibodies at a median of 18 days and 26 days after the second dose, respectively. PLWH had lower levels of RDB-IgG than controls, but their immune sera had neutralizing activities against SARS-CoV-2 pseudo-virus similar to controls. A drop in CD4+ T-cell counts was reported after the first and second vaccine and at 4 months post vaccination in PLWH. CD4+ T-cell counts decreased from a geometric mean of 700 (97% CI, 648–757) cell/µL to 531 (429–657) following the first doses and 633 (95% CI, 588–683) cell/µL after the second doses of vaccine (*p* < 0.01, relative to baseline before vaccination). A similar decrease in CD4+ T-cell counts was not reported in other studies concerning different types of vaccinations. A transient increase in HIV RNA viral load was detected in three individuals immediately after vaccination, but HIV RNA viral load remained <100 copies/mL. The blips are not to be considered as a viral failure and they occur in a quarter of PLWH under ART. However, they indicate an HIV activation and its potential reservoir increase [111]. None of the patients developed an immediate or delayed-type hypersensitivity reaction [110].

Another study investigated the levels of anti-spike and neutralizing antibodies in relation to CD4+ T-cell counts or CD4:CD8+T-cell ratios in 140 PLWH following vaccination with BNT162b2 in a prime/boost regimen. The time interval between the first and the second vaccination was 29 days. All PLWH had a viral load <200 HIV-1 RNA copies/mL and 96.5% had a viral load <50 copies/mL. The 88 patients tested after one vaccination had a mean CD4+ T-cell count/μL of 716 (151–1558) while the 52 individuals tested after the second dose had a CD4+ T-cell count/μL of 577 (45–1106). Anti-S IgG became detectable in most patients from day 10 after priming and increased over time. The increase in IgG concentration was associated with an increase in antibody-inhibitory activity in a virus surrogate neutralization test. PLWH with CD4+ T-cell counts below 500 cells/μL and those with CD4+ T-cell counts above 500 cells/μL had similar levels of anti-S IgG. Within variance, anti-S IgG and IgA or inhibitory activity were significantly higher in PLWH with CD4:CD8 ratios > 0.5 compared with those with CD4:CD8 ratios < 0.5 and this association was lost after BNT162b2 booster vaccination. In addition, PLWH produced significantly lower levels of anti-S IgG after prime and boost vaccinations compared with health-care workers [112]. Similarly, the results of the BNT162b vaccination trial including 90 PLWH and 90 controls demonstrated a high rate of seroconversion in PLWH, although the levels of spike IgG antibodies, after two doses of vaccine, were significantly lower in HIV patients than in healthy controls. In this study, all patients were on ART and 86% of them had HIV RNA less than 50 copies/μL, while their CD4+ T-cell count was 565 (280–723) cells/μL [113].

The capacity of the BNT162b2 vaccine to induce both cellular and humoral immune responses in PLWH was investigated in the study by Woldemeskel et al. [114]. In this study, the frequencies of spike-specific IFN-γ-secreting T cells, the titers of spike-binding antibodies and the levels of antibodies able to block SARS-CoV-2 binding to ACE2 were determined in 12 PLWH between 7 and 17 days after the second vaccine dose. The participants had a median age of 52 years, all were on antiretroviral therapy and three had low-level viremia. The median CD4+ T-cell counts were of 913 cell/µL. Included as controls were 17 healthy donors. The analysis of the cellular immune response demonstrated that PLWH and healthy controls had similar numbers of IFN-γ-secreting T cells in unfractionated or CD8+T-cell depleted PBMC. Additionally, the breadth and the specificity of the T-cell responses were comparable in the two groups.

In addition, PLWH and healthy controls developed similar titers of SARS-CoV-2 spike-binding antibodies and similar levels of neutralizing antibodies to the vaccine strain spike protein or to spike variants including alpha (B.1.1.7), beta (B.1.351) and gamma (P.1) strains [114]. More recently, the humoral immune response elicited by the mRNA-1273 vaccine was analyzed in a cohort of 71 PLWH who received two doses of vaccine with a time interval of 28 days. All participants were receiving suppressive ART and they had a median HIV viral load <50 copies/mL and a median CD4+ T-cell count of 747.0 cells per μL (IQR 593–942). Evaluation of the humoral immune response 28 days after the second dose showed that PLWH had similar anti-S titers and neutralizing antibody activity to individuals with no HIV infection [115].

## 7. Specific/Potential Side Effects of SARS-CoV-2 Vaccines in People Living with HIV (PLWH)

The side effects of SARS-CoV-2 vaccines described in the general population have also been observed in PLWH (see Section 4.3).

However, it is worth bearing in mind that an activation of HIV-infected CD4+ T cells has been observed in vaccinated PLWH. Indeed, an increase in HIV RNA viral load was observed in the period immediately after the administration of the SARS-CoV-2 vaccine, concomitantly with a decrease in absolute CD4+ T-cell counts [110]. Since HIV is a retrovirus and therefore has a reverse transcriptase, the question is whether, following recombination events, the messenger RNA encoding the spike protein of SARS-CoV-2 could be included in the proviral DNA, integrated into the human genome and co-packaged in retroviral progenies.

This question was posed by Tombacz I et al. [116]. As is well known, RNA is not able to integrate within the DNA of the host genome unless it is retro-transcribed by viral retro-transcriptases such as those of HIV or by those produced by endogenous retrotransposons.

**Figure 1:**
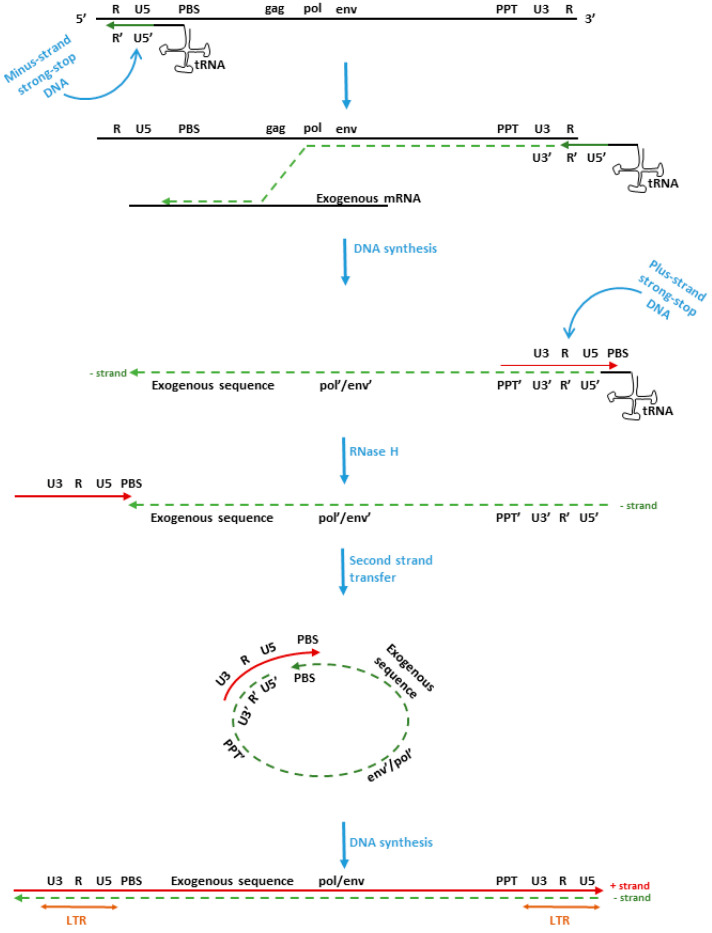
Process of reverse transcription of retroviral DNA: RNA = thin black line, hyphen-line = minus (−) strand DNA, bold, dark color = plus (+) strand DNA. PPT, polyuridine tract, which is resistant to RNase H degradation. PBS, primer binding site; RNase H, ribonuclease H, an enzyme specific for RNA strand: DNA duplexes. During the synthesis of minus (−) strand DNA (dashed-line) there is a possibility of nonhomologous recombination.

To understand how and whether this is possible, we give a brief description of the retrovirus replication cycle (Figure 1). Retroviruses have an RNA genome and within each virion there are two copies of HIV RNA (diploid genome). To replicate, however, the virus requires an intermediate double-stranded DNA that integrates in the form of a provirus in the host’s genome [117]. Recombination takes place during the reverse transcription step and it is estimated that homologous recombination occurs at high frequency (ranging from 10 to 30% for each cycle of replication). According to the dynamic copy choice model, RT can switch between the two co-packaged RNAs using portions of each RNA as a template to generate chimeric DNA-containing sequences from each of the two genomic RNAs during minus-strand DNA synthesis [118].

Nevertheless, nonhomologous recombination can also occur between retrovirus and exogenous RNA. Hajjar and colleagues used the SE21Q1b cell line, which is able to randomly package cellular mRNA into retroviral particles, to study the potential recombination between leukosis viral genomic RNA and neo-containing RNA devoid of retroviral sequences. The *neo* RNA was efficiently packaged into SE21Q1B virions. In most cases, the *neo* RNA was reverse transcribed to the 5′ end and incorporated into the new proviral DNA. The authors hypothesized a jump to the 3′ end of the minus-sense DNA without any homology, although in normal reverse transcription, the primer binding site provides homology for this jump to occur [119].

On the other hand, the Sarbecoronavirus subgenus, which includes SARS-CoV-2, shows high recombination frequency, especially in the spike protein [120,121,122]. A recent study, which was carried out by applying a genome-wide approach to examine polymorphisms, revealed that recombination represents the cause of around 40% of polymorphisms in the coronavirus viral population. Recombinant exchanges were found to be located randomly along the coronavirus genome, even though they were concentrated in regions involved in the interaction with host cells [123].

The high recombination rate is promoted by the presence of subgenomic RNAs, which are generated during replication and which allow template switching [124]. Coronavirus RNA fragments which cannot replicate themselves can be involved in RNA recombination. In nonhomologous recombination, a replicating RNA can recombine with a nonreplicating RNA (e.g., cellular RNA). These experimental data justify the question regarding whether HIV can give rise to recombination events between nonreplicating RNA fragments of the SARS-CoV-2 vaccine and the HIV genome. Currently, experimental evidence that confirm or exclude these scenarios is lacking.

Concerning the AdV vector, Adenoviruses are known to undergo recombination during infection. Phylogenetic analysis revealed that serotype 16 is a product of recombination between sequences of species B and E [125]. Moreover, experimental data demonstrated that AdV12 integrated quickly after the infection of BHK21 hamster cells and that the integration sites are located along the whole human genome [126]. As previously mentioned, in immunosuppressed subjects, adenoviruses can cause chronic infections. Considering the wide tropism of AdVs, the possibility of recombination between the adenoviral vaccine and an AdV strain which causes chronic infection cannot be excluded. Potential alteration in transcriptional profiles should be taken into consideration and experimental data aimed to clarify this aspect are needed. In addition, it should be considered that results from trials of HIV vaccines based on the AdV5 vector found that Ad5 seropositive men were at elevated risk of acquiring HIV-1 during the first 18 months after vaccination [127]. It has been hypothesized that the AdV5 immunocomplex can activate the dendritic-cell–T-cell axis with an increase in HIV viral replication and T-cell susceptibility to HIV infection [128]. In a consensus conference sponsored by the NIH, experts discouraged the use of the AdV5 viral vector in the development of vaccines that might be administered to PLWH [127].

## 8. Conclusions

The data reported in this review highlight that the mortality of PLWH caused by SARS-CoV-2 infection is influenced by the same cofactors observed in HIV-negative subjects, including, for example, age, obesity and *M. tuberculosis* infection status. The humoral immune response, elicited by mRNA and adenoviral-vector vaccines in PLWH is similar to that induced in individuals without HIV infection [107,114]. Nevertheless, these data refer to a short period of observation after vaccination, and there are no data on either the durability of the immune response induced in PLWH or on the degree of protection in the long term. Furthermore, few studies have measured the T-cell response induced by vaccination, its durability and its efficacy against viral variants [114]. In addition, since the majority of data from clinical trials refer to PLWH with good CD4+ T-cell counts, which are sometimes comparable to those of individuals with no HIV infection, the protection period provided by vaccination to PLWH with CD4+ T-cell counts < 200 cells/µL might be shorter than that observed in subjects with CD4+ T-cell counts > 500 cells/µL, and therefore the vaccination should be carried out at shorter intervals. Concerning safety, the same adverse effects have been reported in HIV-negative subjects, for which seropositivity does not seem to represent any further risk factor for anti-COVID-19 vaccination.

In conclusion, hesitancy [129] in vaccinating PLWH subjects appears to be unjustified, while greater certainty is needed regarding the effective duration of vaccine protection in order to correctly define the timing of administration of the booster doses.

## References

[1] WHO Coronavirus (COVID-19) Dashboard. https://covid19.who.int.

[2] HAdV Working Group. http://hadvwg.gmu.edu/.

[3] Gao J., Mese K., Bunz O., Ehrhardt A. (2019). State-of-the-art Human Adenovirus Vectorology for Therapeutic Approaches. FEBS Lett..

[4] Crenshaw B.J., Jones L.B., Bell C.R., Kumar S., Matthews Q.L. (2019). Perspective on Adenoviruses: Epidemiology, Pathogenicity, and Gene Therapy. Biomedicines.

[5] Rauch S., Jasny E., Schmidt K.E., Petsch B. (2018). New Vaccine Technologies to Combat Outbreak Situations. Front. Immunol..

[6] Lee C.S., Bishop E.S., Zhang R., Yu X., Farina E.M., Yan S., Zhao C., Zheng Z., Shu Y., Wu X. (2017). Adenovirus-Mediated Gene Delivery: Potential Applications for Gene and Cell-Based Therapies in the New Era of Personalized Medicine. Genes Dis..

[7] Zhang Y., Bergelson J.M. (2005). Adenovirus Receptors. J. Virol..

[8] Baker A.T., Greenshields-Watson A., Coughlan L., Davies J.A., Uusi-Kerttula H., Cole D.K., Rizkallah P.J., Parker A.L. (2019). Diversity within the Adenovirus Fiber Knob Hypervariable Loops Influences Primary Receptor Interactions. Nat. Commun..

[9] Chen S., Tian X. (2018). Vaccine Development for Human Mastadenovirus. J. Thorac. Dis..

[10] Jobran S., Kattan R., Shamaa J., Marzouqa H., Hindiyeh M. (2018). Adenovirus Respiratory Tract Infections in Infants: A Retrospective Chart-Review Study. Lancet.

[11] Radke J.R., Cook J.L. (2018). Human Adenovirus Infections: Update and Consideration of Mechanisms of Viral Persistence. Curr. Opin. Infect. Dis..

[12] Cook J., Radke J. (2017). Mechanisms of Pathogenesis of Emerging Adenoviruses. F1000Research.

[13] Lamson D.M., Kajon A., Shudt M., Girouard G., St. George K. (2017). Detection and Genetic Characterization of Adenovirus Type 14 Strain in Students with Influenza-Like Illness, New York, USA, 2014–2015. Emerg. Infect. Dis..

[14] https://www.cdc.gov/mmwr/preview/mmwrhtml/mm5645a1.htm.

[15] Louie J.K., Kajon A.E., Holodniy M., Guardia-LaBar L., Lee B., Petru A.M., Hacker J.K., Schnurr D.P. (2008). Severe Pneumonia Due to Adenovirus Serotype 14: A New Respiratory Threat?. Clin. Infect. Dis..

[16] de Mezerville M.H., Tellier R., Richardson S., Hébert D., Doyle J., Allen U. (2006). Adenoviral Infections in Pediatric Transplant Recipients: A Hospital-Based Study. Pediatr. Infect. Dis. J..

[17] Garnett C.T., Erdman D., Xu W., Gooding L.R. (2002). Prevalence and Quantitation of Species C Adenovirus DNA in Human Mucosal Lymphocytes. J. Virol..

[18] Roy S., Calcedo R., Medina-Jaszek A., Keough M., Peng H., Wilson J.M. (2011). Adenoviruses in Lymphocytes of the Human Gastro-Intestinal Tract. PLoS ONE.

[19] Kosulin K., Geiger E., Vécsei A., Huber W.-D., Rauch M., Brenner E., Wrba F., Hammer K., Innerhofer A., Pötschger U. (2016). Persistence and Reactivation of Human Adenoviruses in the Gastrointestinal Tract. Clin. Microbiol. Infect..

[20] Tebruegge M., Curtis N. (2012). Adenovirus: An Overview for Pediatric Infectious Diseases Specialists. Pediatr. Infect. Dis. J..

[21] Adeyemi O.A., Yeldandi A.V., Ison M.G. (2008). Fatal Adenovirus Pneumonia in a Person with AIDS and Burkitt Lymphoma: A Case Report and Review of the Literature. AIDS Read..

[22] Nebbia G., Chawla A., Schutten M., Atkinson C., Raza M., Johnson M., Geretti A. (2005). Adenovirus Viraemia and Dissemination Unresponsive to Antiviral Therapy in Advanced HIV-1 Infection. AIDS.

[23] Heemskerk B., van Vreeswijk T., Veltrop-Duits L.A., Sombroek C.C., Franken K., Verhoosel R.M., Hiemstra P.S., van Leeuwen D., Ressing M.E., Toes R.E.M. (2006). Adenovirus-Specific CD4 + T Cell Clones Recognizing Endogenous Antigen Inhibit Viral Replication In Vitro through Cognate Interaction. J. Immunol..

[24] Shirali G.S., Ni J., Chinnock R.E., Johnston J.K., Rosenthal G.L., Bowles N.E., Towbin J.A. (2001). Association of Viral Genome with Graft Loss in Children after Cardiac Transplantation. N. Engl. J. Med..

[25] McGrath D., Falagas M.E., Freeman R., Rohrer R., Fairchild R., Colbach C., Snydman D.R. (1998). Adenovirus Infection in Adult Orthotopic Liver Transplant Recipients: Incidence and Clinical Significance. J. Infect. Dis..

[26] Öhrmalm L., Smedman C., Wong M., Broliden K., Tolfvenstam T., Norbeck O. (2013). Decreased functional T lymphocyte-mediated cytokine responses in patients with chemotherapy-induced neutropenia. J. Intern. Med..

[27] Verma R., Foster R.E., Horgan K., Mounsey K., Nixon H., Smalle N., Hughes T.A., Carter C.R.D. (2016). Lymphocyte Depletion and Repopulation after Chemotherapy for Primary Breast Cancer. Breast Cancer Res..

[28] Peng B., Wang L.R., Gómez-Román V.R., Davis-Warren A., Montefiori D.C., Kalyanaraman V.S., Venzon D., Zhao J., Kan E., Rowell T.J. (2005). Replicating Rather than Nonreplicating Adenovirus-Human Immunodeficiency Virus Recombinant Vaccines Are Better at Eliciting Potent Cellular Immunity and Priming High-Titer Antibodies. J. Virol..

[29] Wold W., Toth K. (2014). Adenovirus Vectors for Gene Therapy, Vaccination and Cancer Gene Therapy. Curr. Gene Ther..

[30] Tan W.G., Jin H.-T., West E.E., Penaloza-MacMaster P., Wieland A., Zilliox M.J., McElrath M.J., Barouch D.H., Ahmed R. (2013). Comparative Analysis of Simian Immunodeficiency Virus Gag-Specific Effector and Memory CD8 + T Cells Induced by Different Adenovirus Vectors. J. Virol..

[31] Humphreys I.R., Sebastian S. (2018). Novel Viral Vectors in Infectious Diseases. Immunology.

[32] Mennechet F.J.D., Paris O., Ouoba A.R., Salazar Arenas S., Sirima S.B., Takoudjou Dzomo G.R., Diarra A., Traore I.T., Kania D., Eichholz K. (2019). A Review of 65 Years of Human Adenovirus Seroprevalence. Expert Rev. Vaccines.

[33] Kostense S., Koudstaal W., Sprangers M., Weverling G.J., Penders G., Helmus N., Vogels R., Bakker M., Berkhout B., Havenga M. (2004). Adenovirus Types 5 and 35 Seroprevalence in AIDS Risk Groups Supports Type 35 as a Vaccine Vector. AIDS.

[34] Feng L., Wang Q., Shan C., Yang C., Feng Y., Wu J., Liu X., Zhou Y., Jiang R., Hu P. (2020). An Adenovirus-Vectored COVID-19 Vaccine Confers Protection from SARS-COV-2 Challenge in Rhesus Macaques. Nat. Commun..

[35] Wang N., Shang J., Jiang S., Du L. (2020). Subunit Vaccines Against Emerging Pathogenic Human Coronaviruses. Front. Microbiol..

[36] Zhu F.-C., Li Y.-H., Guan X.-H., Hou L.-H., Wang W.-J., Li J.-X., Wu S.-P., Wang B.-S., Wang Z., Wang L. (2020). Safety, Tolerability, and Immunogenicity of a Recombinant Adenovirus Type-5 Vectored COVID-19 Vaccine: A Dose-Escalation, Open-Label, Non-Randomised, First-in-Human Trial. Lancet.

[37] Zhu F.-C., Guan X.-H., Li Y.-H., Huang J.-Y., Jiang T., Hou L.-H., Li J.-X., Yang B.-F., Wang L., Wang W.-J. (2020). Immunogenicity and Safety of a Recombinant Adenovirus Type-5-Vectored COVID-19 Vaccine in Healthy Adults Aged 18 Years or Older: A Randomised, Double-Blind, Placebo-Controlled, Phase 2 Trial. Lancet.

[38] Sadoff J., Le Gars M., Shukarev G., Heerwegh D., Truyers C., de Groot A.M., Stoop J., Tete S., Van Damme W., Leroux-Roels I. (2021). Interim Results of a Phase 1-2a Trial of Ad26.COV2.S Covid-19 Vaccine. N. Engl. J. Med..

[39] Logunov D.Y., Dolzhikova I.V., Zubkova O.V., Tukhvatulin A.I., Shcheblyakov D.V., Dzharullaeva A.S., Grousova D.M., Erokhova A.S., Kovyrshina A.V., Botikov A.G. (2020). Safety and Immunogenicity of an RAd26 and RAd5 Vector-Based Heterologous Prime-Boost COVID-19 Vaccine in Two Formulations: Two Open, Non-Randomised Phase 1/2 Studies from Russia. Lancet.

[40] Voysey M., Clemens S.A.C., Madhi S.A., Weckx L.Y., Folegatti P.M., Aley P.K., Angus B., Baillie V.L., Barnabas S.L., Bhorat Q.E. (2021). Safety and Efficacy of the ChAdOx1 NCoV-19 Vaccine (AZD1222) against SARS-CoV-2: An Interim Analysis of Four Randomised Controlled Trials in Brazil, South Africa, and the UK. Lancet.

[41] Hodgson J. (2020). The Pandemic Pipeline. Nat. Biotechnol..

[42] Karikó K., Buckstein M., Ni H., Weissman D. (2005). Suppression of RNA Recognition by Toll-like Receptors: The Impact of Nucleoside Modification and the Evolutionary Origin of RNA. Immunity.

[43] Karikó K., Muramatsu H., Welsh F.A., Ludwig J., Kato H., Akira S., Weissman D. (2008). Incorporation of Pseudouridine Into MRNA Yields Superior Nonimmunogenic Vector With Increased Translational Capacity and Biological Stability. Mol. Ther..

[44] Anderson B.R., Muramatsu H., Jha B.K., Silverman R.H., Weissman D., Kariko K. (2011). Nucleoside Modifications in RNA Limit Activation of 2’-5’-Oligoadenylate Synthetase and Increase Resistance to Cleavage by RNase L. Nucleic Acids Res..

[45] Devarkar S.C., Wang C., Miller M.T., Ramanathan A., Jiang F., Khan A.G., Patel S.S., Marcotrigiano J. (2016). Structural Basis for M7G Recognition and 2′-O-Methyl Discrimination in Capped RNAs by the Innate Immune Receptor RIG-I. Proc. Natl. Acad. Sci. USA.

[46] Stepinski J., Waddell C., Stolarski R., Darzynkiewicz E., Rhoads R.E. (2001). Synthesis and Properties of MRNAs Containing the Novel “Anti-Reverse” Cap Analogs 7-Methyl(3′-O-Methyl)GpppG and 7-Methyl (3′-Deoxy)GpppG. RNA.

[47] Jemielity J., Fowler T., Zuberek J., Stepinski J., Lewdorowicz M., Niedzwiecka A., Stolarski R., Darzynkiewicz E., Rhoads R.E. (2003). Novel “Anti-Reverse” Cap Analogs with Superior Translational Properties. RNA.

[48] Grudzien-Nogalska E., Stepinski J., Jemielity J., Zuberek J., Stolarski R., Rhoads R.E., Darzynkiewicz E. (2007). Synthesis of Anti-Reverse Cap Analogs (ARCAs) and Their Applications in MRNA Translation and Stability. Methods in Enzymology.

[49] Vaidyanathan S., Azizian K.T., Haque A.K.M.A., Henderson J.M., Hendel A., Shore S., Antony J.S., Hogrefe R.I., Kormann M.S.D., Porteus M.H. (2018). Uridine Depletion and Chemical Modification Increase Cas9 MRNA Activity and Reduce Immunogenicity without HPLC Purification. Mol. Ther.—Nucleic Acids.

[50] Pardi N., Hogan M.J., Weissman D. (2020). Recent Advances in MRNA Vaccine Technology. Curr. Opin. Immunol..

[51] Leppek K., Das R., Barna M. (2018). Functional 5’ UTR MRNA Structures in Eukaryotic Translation Regulation and How to Find Them. Nat. Rev. Mol. Cell Biol..

[52] Tanguay R.L., Gallie D.R. (1996). Translational Efficiency Is Regulated by the Length of the 3′ Untranslated Region. Mol. Cell. Biol..

[53] Gray N.K., Wickens M. (1998). Control of translation initiation in animals. Annu. Rev. Cell Dev. Biol..

[54] Kozak M. (1987). At Least Six Nucleotides Preceding the AUG Initiator Codon Enhance Translation in Mammalian Cells. J. Mol. Biol..

[55] Ferizi M., Leonhardt C., Meggle C., Aneja M.K., Rudolph C., Plank C., Rädler J.O. (2015). Stability Analysis of Chemically Modified MRNA Using Micropattern-Based Single-Cell Arrays. Lab Chip.

[56] Orlandini von Niessen A.G., Poleganov M.A., Rechner C., Plaschke A., Kranz L.M., Fesser S., Diken M., Löwer M., Vallazza B., Beissert T. (2019). Improving MRNA-Based Therapeutic Gene Delivery by Expression-Augmenting 3′ UTRs Identified by Cellular Library Screening. Mol. Ther..

[57] Linares-Fernández S., Lacroix C., Exposito J.-Y., Verrier B. (2020). Tailoring MRNA Vaccine to Balance Innate/Adaptive Immune Response. Trends Mol. Med..

[58] Grier A.E., Burleigh S., Sahni J., Clough C.A., Cardot V., Choe D.C., Krutein M.C., Rawlings D.J., Jensen M.C., Scharenberg A.M. (2016). PEVL: A Linear Plasmid for Generating MRNA IVT Templates With Extended Encoded Poly(A) Sequences. Mol. Ther.—Nucleic Acids.

[59] Park J.-E., Yi H., Kim Y., Chang H., Kim V.N. (2016). Regulation of Poly(A) Tail and Translation during the Somatic Cell Cycle. Mol. Cell.

[60] Karikó K., Muramatsu H., Ludwig J., Weissman D. (2011). Generating the Optimal MRNA for Therapy: HPLC Purification Eliminates Immune Activation and Improves Translation of Nucleoside-Modified, Protein-Encoding MRNA. Nucleic Acids Res..

[61] Baiersdörfer M., Boros G., Muramatsu H., Mahiny A., Vlatkovic I., Sahin U., Karikó K. (2019). A Facile Method for the Removal of DsRNA Contaminant from In Vitro-Transcribed MRNA. Mol. Ther.—Nucleic Acids.

[62] Pardi N., Tuyishime S., Muramatsu H., Kariko K., Mui B.L., Tam Y.K., Madden T.D., Hope M.J., Weissman D. (2015). Expression Kinetics of Nucleoside-Modified MRNA Delivered in Lipid Nanoparticles to Mice by Various Routes. J. Control. Release Off. J. Control. Release Soc..

[63] Pardi N., Hogan M.J., Porter F.W., Weissman D. (2018). MRNA Vaccines—A New Era in Vaccinology. Nat. Rev. Drug Discov..

[64] Semple S.C., Akinc A., Chen J., Sandhu A.P., Mui B.L., Cho C.K., Sah D.W.Y., Stebbing D., Crosley E.J., Yaworski E. (2010). Rational Design of Cationic Lipids for SiRNA Delivery. Nat. Biotechnol..

[65] Kowalski P.S., Rudra A., Miao L., Anderson D.G. (2019). Delivering the Messenger: Advances in Technologies for Therapeutic MRNA Delivery. Mol. Ther..

[66] Lonez C., Vandenbranden M., Ruysschaert J.-M. (2012). Cationic Lipids Activate Intracellular Signaling Pathways. Adv. Drug Deliv. Rev..

[67] Hoffmann M., Kleine-Weber H., Schroeder S., Krüger N., Herrler T., Erichsen S., Schiergens T.S., Herrler G., Wu N.-H., Nitsche A. (2020). SARS-CoV-2 Cell Entry Depends on ACE2 and TMPRSS2 and Is Blocked by a Clinically Proven Protease Inhibitor. Cell.

[68] Folegatti P.M., Ewer K.J., Aley P.K., Angus B., Becker S., Belij-Rammerstorfer S., Bellamy D., Bibi S., Bittaye M., Clutterbuck E.A. (2020). Safety and Immunogenicity of the ChAdOx1 NCoV-19 Vaccine against SARS-CoV-2: A Preliminary Report of a Phase 1/2, Single-Blind, Randomised Controlled Trial. Lancet.

[69] Ewer K.J., Barrett J.R., Belij-Rammerstorfer S., Sharpe H., Makinson R., Morter R., Flaxman A., Wright D., Bellamy D., Bittaye M. (2021). T Cell and Antibody Responses Induced by a Single Dose of ChAdOx1 NCoV-19 (AZD1222) Vaccine in a Phase 1/2 Clinical Trial. Nat. Med..

[70] Barrett J.R., Belij-Rammerstorfer S., Dold C., Ewer K.J., Folegatti P.M., Gilbride C., Halkerston R., Hill J., Jenkin D., Stockdale L. (2021). Phase 1/2 Trial of SARS-CoV-2 Vaccine ChAdOx1 NCoV-19 with a Booster Dose Induces Multifunctional Antibody Responses. Nat. Med..

[71] Ramasamy M.N., Minassian A.M., Ewer K.J., Flaxman A.L., Folegatti P.M., Owens D.R., Voysey M., Aley P.K., Angus B., Babbage G. (2021). Safety and Immunogenicity of ChAdOx1 NCoV-19 Vaccine Administered in a Prime-Boost Regimen in Young and Old Adults (COV002): A Single-Blind, Randomised, Controlled, Phase 2/3 Trial. Lancet.

[72] Walsh E.E., Frenck R.W., Falsey A.R., Kitchin N., Absalon J., Gurtman A., Lockhart S., Neuzil K., Mulligan M.J., Bailey R. (2020). Safety and Immunogenicity of Two RNA-Based Covid-19 Vaccine Candidates. N. Engl. J. Med..

[73] Sahin U., Muik A., Vogler I., Derhovanessian E., Kranz L.M., Vormehr M., Quandt J., Bidmon N., Ulges A., Baum A. (2021). BNT162b2 Vaccine Induces Neutralizing Antibodies and Poly-Specific T Cells in Humans. Nature.

[74] Frenck R.W., Klein N.P., Kitchin N., Gurtman A., Absalon J., Lockhart S., Perez J.L., Walter E.B., Senders S., Bailey R. (2021). Safety, Immunogenicity, and Efficacy of the BNT162b2 Covid-19 Vaccine in Adolescents. N. Engl. J. Med..

[75] Jackson L.A., Anderson E.J., Rouphael N.G., Roberts P.C., Makhene M., Coler R.N., McCullough M.P., Chappell J.D., Denison M.R., Stevens L.J. (2020). An MRNA Vaccine against SARS-CoV-2—Preliminary Report. N. Engl. J. Med..

[76] Anderson E.J., Rouphael N.G., Widge A.T., Jackson L.A., Roberts P.C., Makhene M., Chappell J.D., Denison M.R., Stevens L.J., Pruijssers A.J. (2020). Safety and Immunogenicity of SARS-CoV-2 MRNA-1273 Vaccine in Older Adults. N. Engl. J. Med..

[77] Widge A.T., Rouphael N.G., Jackson L.A., Anderson E.J., Roberts P.C., Makhene M., Chappell J.D., Denison M.R., Stevens L.J., Pruijssers A.J. (2021). Durability of Responses after SARS-CoV-2 MRNA-1273 Vaccination. N. Engl. J. Med..

[78] Polack F.P., Thomas S.J., Kitchin N., Absalon J., Gurtman A., Lockhart S., Perez J.L., Pérez Marc G., Moreira E.D., Zerbini C. (2020). Safety and Efficacy of the BNT162b2 MRNA Covid-19 Vaccine. N. Engl. J. Med..

[79] Kircheis R. (2021). Coagulopathies after Vaccination against SARS-CoV-2 May Be Derived from a Combined Effect of SARS-CoV-2 Spike Protein and Adenovirus Vector-Triggered Signaling Pathways. Int. J. Mol. Sci..

[80] Greinacher A., Thiele T., Warkentin T.E., Weisser K., Kyrle P.A., Eichinger S. (2021). Thrombotic Thrombocytopenia after ChAdOx1 NCov-19 Vaccination. N. Engl. J. Med..

[81] Schultz N.H., Sørvoll I.H., Michelsen A.E., Munthe L.A., Lund-Johansen F., Ahlen M.T., Wiedmann M., Aamodt A.-H., Skattør T.H., Tjønnfjord G.E. (2021). Thrombosis and Thrombocytopenia after ChAdOx1 NCoV-19 Vaccination. N. Engl. J. Med..

[82] Scully M., Singh D., Lown R., Poles A., Solomon T., Levi M., Goldblatt D., Kotoucek P., Thomas W., Lester W. (2021). Pathologic Antibodies to Platelet Factor 4 after ChAdOx1 NCoV-19 Vaccination. N. Engl. J. Med..

[83] Anand P., Stahel V.P. (2021). The Safety of Covid-19 MRNA Vaccines: A Review. Patient Saf. Surg..

[84] Blanco J.L., Ambrosioni J., Garcia F., Martínez E., Soriano A., Mallolas J., Miro J.M. (2020). COVID-19 in Patients with HIV: Clinical Case Series. Lancet HIV.

[85] Okoh A.K., Sossou C., Dangayach N.S., Meledathu S., Phillips O., Raczek C., Patti M., Kang N., Hirji S.A., Cathcart C. (2020). Coronavirus Disease 19 in Minority Populations of Newark, New Jersey. Int. J. Equity Health.

[86] Cooper T., Woodward B., Alom S., Harky A. (2020). Coronavirus Disease 2019 (COVID-19) Outcomes in HIV/AIDS Patients: A Systematic Review. HIV Med..

[87] Bhaskaran K., Rentsch C.T., MacKenna B., Schultze A., Mehrkar A., Bates C.J., Eggo R.M., Morton C.E., Bacon S.C.J., Inglesby P. (2021). HIV infection and COVID-19 death: A population-based cohort analysis of UK primary care data and linked national death registrations within the OpenSAFELY platform. Lancet HIV.

[88] Vizcarra P., Pérez-Elías M.J., Quereda C., Moreno A., Vivancos M.J., Dronda F., Casado J.L., Moreno S., Pérez-Elías M.J., Fortún J. (2020). Description of COVID-19 in HIV-Infected Individuals: A Single-Centre, Prospective Cohort. Lancet HIV.

[89] Dandachi D., Geiger G., Montgomery M.W., Karmen-Tuohy S., Golzy M., Antar A.A.R., Llibre J.M., Camazine M., Díaz-De Santiago A., Carlucci P.M. (2021). Characteristics, Comorbidities, and Outcomes in a Multicenter Registry of Patients With Human Immunodeficiency Virus and Coronavirus Disease 2019. Clin. Infect. Dis. Off. Publ. Infect. Dis. Soc. Am..

[90] Nomah D.K., Reyes-Urueña J., Díaz Y., Moreno S., Aceiton J., Bruguera A., Vivanco-Hidalgo R.M., Llibre J.M., Domingo P., Falcó V. (2021). Sociodemographic, Clinical, and Immunological Factors Associated with SARS-CoV-2 Diagnosis and Severe COVID-19 Outcomes in People Living with HIV: A Retrospective Cohort Study. Lancet HIV.

[91] Vaughn V.M., Gandhi T.N., Petty L.A., Patel P.K., Prescott H.C., Malani A.N., Ratz D., McLaughlin E., Chopra V., Flanders S.A. (2021). Empiric Antibacterial Therapy and Community-Onset Bacterial Coinfection in Patients Hospitalized With Coronavirus Disease 2019 (COVID-19): A Multi-Hospital Cohort Study. Clin. Infect. Dis..

[92] Nagarakanti S.R., Okoh A.K., Grinberg S., Bishburg E. (2021). Clinical Outcomes of Patients with COVID-19 and HIV Coinfection. J. Med. Virol..

[93] Wang M., Luo L., Bu H., Xia H. (2020). One Case of Coronavirus Disease 2019 (COVID-19) in a Patient Co-Infected by HIV with a Low CD4+ T-Cell Count. Int. J. Infect. Dis..

[94] Western Cape Department of Health in Collaboration with the National Institute for Communicable Diseases, South Africa (2021). Risk Factors for Coronavirus Disease 2019 (COVID-19) Death in a Population Cohort Study from the Western Cape Province, South Africa. Clin. Infect. Dis. Off. Publ. Infect. Dis. Soc. Am..

[95] Jacobs G.P., Bhat P., Owiti P., Edwards J.K., Tweya H., Najjemba R. (2017). Did the 2014 Ebola Outbreak in Liberia Affect HIV Testing, Linkage to Care and ART Initiation?. Public Health Action.

[96] Triant V.A. (2013). Cardiovascular Disease and HIV Infection. Curr. HIV/AIDS Rep..

[97] Gervasoni C., Meraviglia P., Riva A., Giacomelli A., Oreni L., Minisci D., Atzori C., Ridolfo A., Cattaneo D. (2020). Clinical Features and Outcomes of Patients With Human Immunodeficiency Virus With COVID-19. Clin. Infect. Dis. Off. Publ. Infect. Dis. Soc. Am..

[98] Byrd K.M., Beckwith C.G., Garland J.M., Johnson J.E., Aung S., Cu-Uvin S., Farmakiotis D., Flanigan T., Gillani F.S., Macias-Gil R. (2020). SARS-CoV-2 and HIV Coinfection: Clinical Experience from Rhode Island, United States. J. Int. AIDS Soc..

[99] Calza L., Colangeli V., Borderi M., Bon I., Borioni A., Volpato F., Re M.C., Viale P. (2020). Weight Gain in Antiretroviral Therapy-Naive HIV-1-Infected Patients Starting a Regimen Including an Integrase Strand Transfer Inhibitor or Darunavir/Ritonavir. Infection.

[100] Guo W., Ming F., Feng Y., Zhang Q., Mo P., Liu L., Gao M., Tang W., Liang K. (2020). Patterns of HIV and SARS-CoV-2 Co-infection in Wuhan, China. J. Int. AIDS Soc..

[101] Gudipati S., Brar I., Murray S., McKinnon J.E., Yared N., Markowitz N. (2020). Descriptive Analysis of Patients Living With HIV Affected by COVID-19. J. Acquir. Immune Defic. Syndr..

[102] Meyerowitz E.A., Kim A.Y., Ard K.L., Basgoz N., Chu J.T., Hurtado R.M., Lee C.K., He W., Minukas T., Nelson S. (2020). Disproportionate Burden of Coronavirus Disease 2019 among Racial Minorities and Those in Congregate Settings among a Large Cohort of People with HIV. AIDS.

[103] Yang X., Sun J., Patel R.C., Zhang J., Guo S., Zheng Q., Olex A.L., Olatosi B., Weissman S.B., Islam J.Y. (2021). Associations between HIV Infection and Clinical Spectrum of COVID-19: A Population Level Analysis Based on US National COVID Cohort Collaborative (N3C) Data. Lancet HIV.

[104] Use of COVID-19 Vaccines in the United States. https://www.cdc.gov/vaccines/covid-19/clinical-considerations/immunocompromised.html.

[105] Frater J., Ewer K.J., Ogbe A., Pace M., Adele S., Adland E., Alagaratnam J., Aley P.K., Ali M., Ansari M.A. (2021). Safety and Immunogenicity of the ChAdOx1 NCoV-19 (AZD1222) Vaccine against SARS-CoV-2 in HIV Infection: A Single-Arm Substudy of a Phase 2/3 Clinical Trial. Lancet HIV.

[106] Access to Antiretroviral Therapy in Africa. https://www.unaids.org/sites/default/files/media_asset/20131219_AccessARTAfricaStatusReportProgresstowards2015Targets_en_0.pdf.

[107] Madhi S.A., Koen A.L., Izu A., Fairlie L., Cutland C.L., Baillie V., Padayachee S.D., Dheda K., Barnabas S.L., Bhorat Q.E. (2021). Safety and Immunogenicity of the ChAdOx1 NCoV-19 (AZD1222) Vaccine against SARS-CoV-2 in People Living with and without HIV in South Africa: An Interim Analysis of a Randomised, Double-Blind, Placebo-Controlled, Phase 1B/2A Trial. Lancet HIV.

[108] Ruddy J.A., Boyarsky B.J., Werbel W.A., Bailey J.R., Karaba A.H., Garonzik-Wang J.M., Segev D.L., Durand C.M. (2021). Safety and Antibody Response to the First Dose of Severe Acute Respiratory Syndrome Coronavirus 2 Messenger RNA Vaccine in Persons with HIV. AIDS.

[109] Ruddy J.A., Boyarsky B.J., Bailey J.R., Karaba A.H., Garonzik-Wang J.M., Segev D.L., Durand C.M., Werbel W.A. (2021). Safety and Antibody Response to Two-Dose SARS-CoV-2 Messenger RNA Vaccination in Persons with HIV. AIDS.

[110] Levy I., Wieder-Finesod A., Litchevsky V., Biber A., Indenbaum V., Olmer L., Huppert A., Mor O., Goldstein M., Levin E.G. (2021). Immunogenicity and Safety of the BNT162b2 MRNA COVID-19 Vaccine in People Living with HIV-1. Clin. Microbiol. Infect. Off. Publ. Eur. Soc. Clin. Microbiol. Infect. Dis..

[111] Sklar P.A., Ward D.J., Baker R.K., Wood K.C., Gafoor Z., Alzola C.F., Moorman A.C., Holmberg S.D., HIV Outpatient Study (HOPS) Investigators (2002). Prevalence and Clinical Correlates of HIV Viremia (‘blips’) in Patients with Previous Suppression below the Limits of Quantification. AIDS.

[112] Jedicke N., Stankov M.V., Cossmann A., Dopfer-Jablonka A., Knuth C., Ahrenstorf G., Ramos G.M., Behrens G.M.N. (2021). Humoral Immune Response Following Prime and Boost BNT162b2 Vaccination in People Living with HIV on Antiretroviral Therapy. HIV Med..

[113] Xu X., Vesterbacka J., Aleman S., Nowak P., COVAXID Study Group (2022). High Seroconversion Rate after Vaccination with MRNA BNT162b2 Vaccine against SARS-CoV-2 among People with HIV—but HIV Viremia Matters?. AIDS.

[114] Woldemeskel B.A., Karaba A.H., Garliss C.C., Beck E.J., Wang K.H., Laeyendecker O., Cox A.L., Blankson J.N. (2021). The BNT162b2 MRNA Vaccine Elicits Robust Humoral and Cellular Immune Responses in People Living with HIV. Clin. Infect. Dis. Off. Publ. Infect. Dis. Soc. Am..

[115] Lombardi A., Butta G.M., Donnici L., Bozzi G., Oggioni M., Bono P., Matera M., Consonni D., Ludovisi S., Muscatello A. (2022). Anti-Spike Antibodies and Neutralising Antibody Activity in People Living with HIV Vaccinated with COVID-19 MRNA-1273 Vaccine: A Prospective Single-Centre Cohort Study. Lancet Reg. Health Eur..

[116] Tombácz I., Weissman D., Pardi N. (2021). Vaccination with Messenger RNA: A Promising Alternative to DNA Vaccination. Methods Mol. Biol..

[117] Varmus H., Brown P., Berg D.E., Howe M.M. (1989). Retroviruses. Mobile DNA.

[118] Hwang C.K., Svarovskaia E.S., Pathak V.K. (2001). Dynamic Copy Choice: Steady State between Murine Leukemia Virus Polymerase and Polymerase-Dependent RNase H Activity Determines Frequency of in Vivo Template Switching. Proc. Natl. Acad. Sci. USA.

[119] Hajjar A.M., Linial M.L. (1993). A Model System for Nonhomologous Recombination between Retroviral and Cellular RNA. J. Virol..

[120] Woo P.C.Y., Lau S.K.P., Yip C.C.Y., Huang Y., Tsoi H.-W., Chan K.-H., Yuen K.-Y. (2006). Comparative Analysis of 22 Coronavirus HKU1 Genomes Reveals a Novel Genotype and Evidence of Natural Recombination in Coronavirus HKU1. J. Virol..

[121] Woo P.C., Lau S.K., Yuen K. (2006). Infectious Diseases Emerging from Chinese Wet-Markets: Zoonotic Origins of Severe Respiratory Viral Infections. Curr. Opin. Infect. Dis..

[122] Woo P.C.Y., Lau S.K.P., Huang Y., Yuen K.-Y. (2009). Coronavirus Diversity, Phylogeny and Interspecies Jumping. Exp. Biol. Med..

[123] Bobay L.-M., O’Donnell A.C., Ochman H. (2020). Recombination Events Are Concentrated in the Spike Protein Region of Betacoronaviruses. PLoS Genet..

[124] Lau S.K.P., Woo P.C.Y., Yip C.C.Y., Tse H., Tsoi H., Cheng V.C.C., Lee P., Tang B.S.F., Cheung C.H.Y., Lee R.A. (2006). Coronavirus HKU1 and Other Coronavirus Infections in Hong Kong. J. Clin. Microbiol..

[125] Ebner K., Pinsker W., Lion T. (2005). Comparative Sequence Analysis of the Hexon Gene in the Entire Spectrum of Human Adenovirus Serotypes: Phylogenetic, Taxonomic, and Clinical Implications. J. Virol..

[126] Doerfler W. (1996). A New Concept in (Adenoviral) Oncogenesis: Integration of Foreign DNA and Its Consequences. Biochim. Biophys. Acta.

[127] Buchbinder S.P., McElrath M.J., Dieffenbach C., Corey L. (2020). Use of Adenovirus Type-5 Vectored Vaccines: A Cautionary Tale. Lancet.

[128] Auclair S., Liu F., Niu Q., Hou W., Churchyard G., Morgan C., Frahm N., Nitayaphan S., Pitisuthithum P., Rerks-Ngarm S. (2018). Distinct Susceptibility of HIV Vaccine Vector-Induced CD4 T Cells to HIV Infection. PLoS Pathog..

[129] Vallée A., Fourn E., Majerholc C., Touche P., Zucman D. (2021). COVID-19 Vaccine Hesitancy among French People Living with HIV. Vaccines.

